# Knowledge, attitudes, and perceived barriers toward guided endodontic access among dental practitioners in Saudi Arabia: a cross-sectional study

**DOI:** 10.1186/s12903-026-08743-3

**Published:** 2026-06-04

**Authors:** Ahmed A. Madfa, Bassam A. Anazi, Abdulmjeed S. Al-Enizy, Sulaiman M. Alshammari, Rawan H. Alshammari, Fai A Alfaleh, Heba A. AlKhairAllah, Abdullah F. Alshammari

**Affiliations:** 1 Department of Restorative Dental Science, College of Dentistry, University of Ha’il, Ha’il, Kingdom of Saudi Arabia; 2 Department of Basic Dental and Medical Science, College of Dentistry, University of Ha’il, Ha’il, Kingdom of Saudi Arabia; 3 Department of endodontics, Qassim Regional Dental Center, Al-Qassim Health Cluster, Qassim, Kingdom of Saudi Arabia; 4 College of Dentistry, University of Ha’il, Ha’il, Kingdom of Saudi Arabia; 5 College of Dentistry, Qassim University, Qassim, Kingdom of Saudi Arabia

**Keywords:** Cone-beam computed tomography, Dental practitioners, Digital dentistry, Endodontics, Guided endodontic access, Technology adoption, Saudi Arabia.

## Abstract

**Background:**

Guided endodontic access (GEA) is an emerging digital technique that integrates cone-beam computed tomography (CBCT) with three-dimensional printing to enable precise and conservative access cavity preparation. It enhances canal localization and reduces procedural errors, particularly in cases with pulp canal calcification or complex root canal anatomy. Despite these advantages, its clinical adoption remains limited. This study aimed to assess the knowledge, attitudes, and perceived barriers toward GEA among dental practitioners in Saudi Arabia and to examine factors influencing its adoption.

**Methods:**

A cross-sectional survey was conducted among dental practitioners actively engaged in clinical practice in Saudi Arabia. Data were collected using a structured, validated electronic questionnaire distributed via professional dental networks. The questionnaire assessed demographic characteristics, awareness and training in GEA, perceived clinical benefits, and barriers to implementation. Descriptive statistics were used to summarize responses, and chi-square tests were applied to evaluate associations between practitioner characteristics and GEA-related knowledge, attitudes, and adoption patterns. Statistical significance was set at *p* < 0.05.

**Results:**

A total of 401 dental practitioners participated in the study. Most respondents (85.5%) reported prior awareness of GEA, and 78.8% indicated having received formal training. Participants demonstrated generally positive attitudes toward GEA, with most agreeing that it improves procedural accuracy, enhances safety, and facilitates management of complex endodontic cases. However, barriers to implementation included high cost, increased procedural time, and the need for specialized training and equipment. Significant associations were observed between GEA knowledge and adoption and practitioners’ gender, clinical experience, and academic qualifications.

**Conclusions:**

Dental practitioners in Saudi Arabia demonstrate high awareness and favorable perceptions of GEA; however, practical barriers continue to limit its widespread adoption. Addressing cost limitations, expanding training opportunities, and integrating digital workflows into dental education may facilitate broader clinical implementation.

**Supplementary Information:**

The online version contains supplementary material available at 10.1186/s12903-026-08743-3.

## Introduction

Accurate and conservative access cavity preparation is essential for successful root canal therapy, as it enables proper canal localization, facilitates cleaning, shaping, and obturation, and preserves tooth structure. Traditional freehand methods rely on two-dimensional radiography and tactile feedback, which may limit effectiveness in challenging clinical situations such as pulp canal calcification, complex root canal morphology, or previously treated teeth. These limitations may result in missed canals, excessive dentin removal, perforations, and reduced long-term tooth survival [1, 2]. Consequently, technological solutions aimed at improving accuracy and reducing iatrogenic errors are increasingly being adopted.

One such innovation is Guided Endodontic Access (GEA), a computer-assisted approach that integrates cone-beam computed tomography (CBCT) with intraoral surface scanning to create three-dimensional guides for precise drilling and virtual planning of access pathways [3, 4]. Studies have demonstrated that both static and dynamic navigation systems provide greater accuracy than conventional techniques in locating calcified canals and managing complex root canal anatomy [5, 6]. Guided access has been shown to reduce dentin loss, preserve structural integrity, and improve canal localization efficiency, potentially enhancing treatment predictability [7, 8]. Clinical evidence also indicates that guided techniques can manage severely calcified or atypically positioned canals while reducing chair time and operator stress [9, 10].

Despite these advantages, GEA remains underutilized among dental practitioners. Barriers include high costs associated with CBCT imaging and 3D printing, concerns about radiation exposure, limited formal training in digital workflows, and insufficient awareness of guided endodontic procedures [11, 12]. Consequently, a gap persists between technological advancements and routine clinical practice, as many practitioners continue to rely on conventional techniques due to familiarity and perceived simplicity [13, 14].

Although the benefits of GEA are well documented, limited evidence exists regarding practitioners’ knowledge, attitudes, and perceived barriers, particularly in general practice settings [15–17]. Furthermore, little is known about how demographic factors such as gender, experience, and qualifications influence adoption [18–20]. Understanding these factors is essential for developing targeted training and facilitating clinical integration. Therefore, this study aimed to evaluate dental practitioners’ knowledge, attitudes, and perceived barriers toward GEA and to examine the influence of demographic variables on its adoption.

## Methods

This study was conducted in accordance with the principles of the Declaration of Helsinki and received ethical approval from the Institutional Research Ethics Committee of the University of Hail, Saudi Arabia (Approval No.: H-2025-874) prior to data collection. Written informed consent was obtained from all participants before participation. Participation was voluntary, and responses were collected anonymously.

The required sample size was calculated using the formula:

$$n =Z^2 P (1-P)/d^2$$

where P is the estimated population proportion (assumed to be 0.5 to maximize sample size when prevalence is unknown), d is the margin of error (0.05), Z is the Z-score corresponding to a 95% confidence interval (1.96), and n is the required sample size. Based on these parameters, the minimum required sample size was calculated to be 384 participants. Ultimately, 401 dentists participated in the study.

The target population consisted of dental practitioners actively engaged in clinical dental practice. Dentists with varying years of clinical experience and professional backgrounds were eligible to participate, including those with undergraduate qualifications, postgraduate or board certifications, and those who had attended workshops or continuing education programs. Individuals who declined participation or did not complete the questionnaire were excluded from the final analysis.

Data were collected using a self-administered, standardized questionnaire designed specifically for this study (Supplementary Table S1). The questionnaire comprised several sections addressing different aspects of the research. It was structured in a logical sequence to minimize response bias, with perception-based Likert-scale items placed at the end of the survey. The first section collected sociodemographic and professional information, including gender, years of clinical experience, professional qualification, and frequency of performing endodontic procedures (e.g., “What is your gender?”, “How many years of clinical experience do you have?”, “What is your highest qualification?”, “How often do you perform endodontic procedures?”). The second section assessed participants’ awareness of and experience with GEA (e.g., “Have you heard of guided endodontic access (GEA)?”, “Have you received any formal training in GEA?”). Subsequent questions evaluated respondents’ self-reported level of knowledge regarding GEA (e.g., “How would you rate your knowledge of GEA?”).

The questionnaire also explored dentists’ perceptions of GEA, including its clinical usefulness and applicability in complex cases (e.g., “GEA improves procedural accuracy and safety”, “GEA is useful in managing calcified canals”). Additionally, participants were asked about potential barriers to GEA use (e.g., “High-cost limits the use of GEA”, “GEA requires additional clinical time and specialized equipment”). The final section evaluated participants’ views on incorporating GEA into dental education and their willingness to adopt the technique in future practice (e.g., “GEA should be included in dental curricula”, “I am willing to use GEA in my clinical practice”).

To ensure content validity and clarity, the questionnaire was reviewed by experts in dental research and endodontics. A pilot study was conducted with a small group of dentists who were not included in the final sample, and their feedback led to minor revisions to improve clarity and relevance. The internal consistency of the questionnaire was assessed using Cronbach’s alpha coefficient, which indicated good reliability (Cronbach’s alpha = 0.93), supporting the consistency of the measured constructs.

Data were collected via an electronic survey distributed through professional dental networks and commonly used online platforms. Participation was optional and anonymous, and submission of the completed questionnaire was considered implicit consent. Prior to analysis, data were checked for accuracy, consistency, and completeness. Records with substantial missing data, incomplete responses, or duplicates were excluded. The dataset was then coded and validated to ensure data integrity.

All statistical analyses were performed using IBM SPSS Statistics software. Descriptive statistics were used to summarize the data, with frequencies and percentages reported for categorical variables. Associations between dentists’ awareness, knowledge, training, and attitudes toward GEA and demographic characteristics (gender, years of experience, and qualification) were assessed using the chi-square test. Statistical significance was set at *p* < 0.05.

## Results

A total of 401 dental practitioners participated. Gender distribution was balanced, with 203 (50.6%) females and 198 (49.3%) males. Most participants had 1–5 years of clinical experience (54.1%), followed by 6–10 years (38.7%), 11–15 years (6.5%), and > 15 years (0.7%). Regarding qualifications, 53.6% held undergraduate degrees, 41.4% were postgraduate or board-certified, and 5.0% had completed continuing education courses or workshops (Table 1).

**Table 1:** Demographic and professional characteristics (*N* = 401)

Variable	Category	*n* (%)
Gender	Male	198 (49.3)
	Female	203 (50.6)
Years of Clinical Experience	1–5 years	217 (54.1)
	6–10 years	155 (38.7)
	11–15 years	26 (6.5)
	> 15 years	3 (0.7)
Qualification	Undergraduate	215 (53.6)
	Postgraduate / Board-certified	166 (41.4)
	Continuing education / Workshops	20 (5.0)
Q4. Do you routinely perform endodontic treatments?	Yes	350 (87.3)
	No	51 (12.7)
Q5. Have you heard of “guided endodontic access (GEA)”?	Yes	343 (85.5)
	No	29 (7.2)
	Unsure	29 (7.2)
Q6. How would you describe guided endodontic access?	CBCT + 3D-printed guide	336 (83.7)
	Computer-controlled access	32 (8.0)
	Manual technique	14 (3.5)
	Unsure	19 (4.7)
Q7. Have you received any formal training (lecture, workshop, or course) about guided endodontic access?	Yes	319 (79.6)
	No	82 (20.4)

Data are presented as frequencies and percentages. No inferential statistical analysis was performed.

A total of 350 individuals (87.3%) reported performing endodontic procedures routinely. Awareness of GEA was high, with 343 participants (85.5%) indicating that they had heard of the technique. Among respondents, 336 (83.7%) correctly identified GEA as the use of a CBCT-guided 3D-printed access guide. Additionally, 319 participants (79.6%) reported having received formal training in GEA (Table 1).

The majority of participants reported moderate to high self-perceived knowledge of GEA: only 12 (3.0%) rated their knowledge as “very poor,” while 159 (42.1%) evaluated their knowledge as “good” and 109 (27.1%) as “excellent” (Table 2). Most respondents either strongly agreed (157; 39.1%) or agreed (184; 45.9%) that GEA enhances procedural accuracy and safety. Similarly, a large proportion recognised its usefulness in complex endodontic cases, with 198 participants (49.4%) agreeing and 115 (28.6%) strongly agreeing. Despite recognising its advantages, many respondents identified cost and time requirements as potential barriers, with 199 (49.6%) agreeing and 103 (25.7%) strongly agreeing that these factors may limit implementation. Nevertheless, willingness to adopt GEA was high, with 106 (26.4%) strongly agreeing and 204 (50.9%) agreeing that they would be willing to use the technique in practice. Similar trends were observed regarding perceptions of GEA as a significant development (50.9% agree; 23.7% strongly agree), inclusion in dental education (49.4% agree; 33.6% strongly agree), and intention to implement GEA in practice, with 307 (76.6%) planning adoption within the next two years (Table 2).

**Table 2:** Self-perceived knowledge and attitudes toward GEA (*N* = 401)

Variable	Category	*n* (%)
Q8. Rate your self-perceived level of knowledge about guided endodontic access:	Very poor	12 (3.0)
	Poor	36 (9.0)
	Moderate	85 (21.2)
	Good	159 (42.1)
	Excellent	109 (27.1)
Q9. GEA improves the accuracy and safety of canal location.	Strongly disagree	6 (1.5)
	Disagree	3 (0.7)
	Neutral	51 (12.7)
	Agree	184 (45.9)
	Strongly agree	157 (39.1)
Q10. GEA should be used routinely in complex or calcified cases.	Strongly disagree	8 (2.0)
	Disagree	12 (3.0)
	Neutral	68 (17.0)
	Agree	198 (49.4)
	Strongly agree	115 (28.6)
Q11. The cost and time required for GEA limit its practical application.	Strongly disagree	4 (1.0)
	Disagree	9 (2.2)
	Neutral	86 (21.4)
	Agree	199 (49.6)
	Strongly agree	103 (25.7)
Q12. I would be willing to adopt GEA if proper training and equipment were available.	Strongly disagree	6 (1.5)
	Disagree	13 (3.2)
	Neutral	72 (18.0)
	Agree	204 (50.9)
	Strongly agree	106 (26.4)
Q13. Guided access represents a major advancement in endodontic practice.	Strongly disagree	5 (1.2)
	Disagree	10 (2.5)
	Neutral	87 (21.7)
	Agree	204 (50.9)
	Strongly agree	95 (23.7)
Q14. I believe undergraduate or continuing education programs should include training on GEA	Strongly disagree	0 (0)
	Disagree	8 (2.0)
	Neutral	60 (14.9)
	Agree	198 (49.4)
	Strongly agree	135 (33.6)
Q15. Do you plan to adopt guided endodontic access in your clinical practice within the next 2 years?	Yes	307 (76.6)
	Unsure	49 (12.2)
	No	25 (6.2)
	Already implemented	20 (5.0)

Data are presented as frequencies and percentages

Several GEA-related characteristics were found to have significant correlations with gender. Male participants were more likely to have formal training in GEA (*p* = 0.022), awareness of the technique (*p* = 0.021), and perform regular endodontics (*p* = 0.007). Additionally, men claimed to know more about GEA (*p* = 0.003). However, gender was not significantly associated with perceptions of accuracy and safety (*p* = 0.928), utility in difficult instances (*p* = 0.259), cost/time constraints (*p* = 0.723), willingness to adopt GEA (*p* = 0.656), or the perception of GEA as a substantial breakthrough (*p* = 0.915) (Table 3; Supplementary Table S2).

**Table 3:** Association between demographics and GEA-related variables

Variable	Gender χ² (df), *p*-value	Years of Experience χ² (df), *p*-value	Qualification χ² (df), *p*-value
Routine endodontic practice	14.17 (4), 0.007	15.884 (8), 0.044	15.561 (4), 0.004
Heard about GEA	11.61 (4), 0.021	25.951 (8), 0.001	63.756 (4), < 0.001
Description of GEA	8.96 (8), 0.346	24.144 (16), 0.086	31.839 (8), < 0.001
Formal training in GEA	11.41 (4), 0.022	38.056 (8), < 0.001	25.081 (4), < 0.001
Self-perceived knowledge	26.42 (10), 0.003	37.573 (20), 0.010	35.123 (10), < 0.001
Accuracy and safety perception	3.09 (8), 0.928	15.383 (16), 0.497	10.897 (8), 0.208
Usefulness in complex cases	12.41 (10), 0.259	35.408 (20), 0.018	36.393 (10), < 0.001
Cost/time limitation	7.03 (10), 0.723	18.952 (20), 0.525	10.918 (10), 0.364
Willingness to adopt	7.73 (10), 0.656	72.180 (20), < 0.001	12.613 (10), 0.246
Major advancement perception	4.62 (10), 0.915	27.962 (20), 0.110	42.402 (10), < 0.001
Inclusion in education	15.90 (8), 0.044	24.117 (16), 0.087	6.203 (8), 0.625
Plan to adopt	1.59 (8), 0.991	36.417 (16), 0.003	36.417 (16), 0.003

These findings may be influenced by differences in access to continuing education, clinical exposure, or engagement with digital technologies. However, as this study did not investigate the underlying causes, these observations should be interpreted with caution. Future studies are recommended to explore these factors and to develop targeted strategies that ensure equitable access to training and technological adoption across genders.

Years of clinical experience were significantly associated with several variables, including routine endodontics (*p* = 0.044), knowledge of GEA (*p* = 0.001), formal training (*p* < 0.001), self-perceived knowledge (*p* = 0.010), perceived utility in complex cases (*p* = 0.018), willingness to adopt GEA (*p* < 0.001), and intention to adopt the technique (*p* = 0.003). By contrast, years of experience did not significantly affect views of accuracy and safety (*p* = 0.497), cost/time constraints (*p* = 0.525), perceptions of GEA as a major breakthrough (*p* = 0.110), or involvement in education (*p* = 0.087) (Table 3; Supplementary Table S3).

Academic qualification also showed significant associations with several variables, including routine endodontic practice (*p* = 0.004), awareness of GEA (*p* < 0.001), accurate description of GEA (*p* < 0.001), formal training (*p* < 0.001), self-perceived knowledge (*p* < 0.001), perceived utility in complex cases (*p* < 0.001), perception of GEA as a major advancement (*p* < 0.001), and intention to adopt the technique (*p* < 0.001). However, academic qualification was not significantly associated with perceptions of accuracy and safety (*p* = 0.208), cost/time constraints (*p* = 0.364), adoption readiness (*p* = 0.246), and participation in schooling (*p* = 0.625) (Table 3; Supplementary Table S4).

## Discussion

The present study evaluated Saudi dentists’ knowledge, attitudes, and perceived barriers regarding the use of GEA. Overall, practitioners demonstrated a high degree of awareness and generally favourable perceptions of its therapeutic advantages and clinical applications. However, its routine use in everyday clinical practice remains limited by several practical challenges. High cost, the need for specialised training, and difficulties integrating the technique into existing clinical workflows were the most frequently reported barriers. These findings suggest that although guided endodontic technologies are increasingly recognised and accepted, addressing these practical limitations is essential to facilitate their wider integration into modern digital dentistry.

The present study found that more than four-fifths of participants were familiar with GEA. This relatively high level of awareness may reflect the growing global emphasis on digital dentistry, as well as the increased familiarity of medical professionals with new technologies through continuing education courses, scientific publications, and professional conferences. The adoption of digital technologies in dentistry has expanded considerably over the past decade, largely driven by advances in digital imaging, computer-assisted treatment planning, and additive manufacturing technologies [21, 22]. These developments have facilitated the integration of guided approaches across multiple dental specialties. Nevertheless, incorporating GEA into routine clinical practice remains challenging. Although many practitioners reported prior exposure or training, its practical application appears limited in clinical settings. Similar trends have been observed with other digital technologies in dentistry, where a gap often exists between awareness and implementation. According to innovation adoption frameworks, clinicians tend to adopt new technologies gradually, depending on perceived clinical benefits, cost, and compatibility with existing workflows. Therefore, without adequate infrastructure, resources, and training opportunities, a technique may not be widely adopted even when its benefits are well understood [23].

Most participants demonstrated an adequate understanding of GEA, particularly regarding access cavity preparation enabled by the integration of digital surface scans, CBCT imaging, and three-dimensional printed guides. This suggests that many practitioners are familiar with the fundamental digital workflow associated with guided endodontic procedures. In guided endodontics, a virtual treatment plan and a patient-specific guide are created by merging CBCT data with intraoral surface scans. This guide enhances the precision and predictability of canal localization by directing the bur along a predefined pathway, particularly in teeth with complex root canal morphology or calcified canals [24, 25].

Participants also expressed generally positive perceptions of GEA, particularly regarding its ability to enhance procedural safety and accuracy during access cavity preparation. This finding is consistent with experimental and clinical research showing that guided techniques can improve canal localization accuracy while preserving dentin structure [12, 25, 26]. A key principle of minimally invasive endodontics is the preservation of tooth structure, which supports improved long-term outcomes and structural integrity. By providing a controlled drilling pathway, minimizing unnecessary dentin removal, and reducing the risk of iatrogenic errors, GEA supports this objective [26]. GEA has proven particularly beneficial in cases involving canal obliteration, complex anatomical variations, or previously treated teeth. In such situations, conventional access preparation can be challenging and may increase the risk of procedural errors, including perforation or excessive dentin removal. By providing a predefined drilling trajectory, GEA improves canal localization accuracy and reduces operator-dependent variability. The effectiveness and reliability of guided techniques, particularly in the management of calcified canals, have been consistently demonstrated in clinical studies and systematic reviews [27].

Despite these advantages, the present study identified several factors limiting the widespread adoption of GEA. Cost was one of the primary barriers, as a guided workflow typically requires CBCT imaging, intraoral scanners, specialized treatment-planning software, and three-dimensional printing technologies. Furthermore, the acquisition and maintenance of this equipment can be financially demanding, particularly for private practices and smaller clinics. Similar barriers have been reported in other areas of digital dentistry, where high infrastructure costs and limited access to advanced technologies often hinder routine clinical implementation [21, 28].

In addition to financial constraints, workflow complexity was identified as another barrier. Compared with conventional access cavity preparation, guided techniques involve additional steps, including digital image acquisition, virtual treatment planning, and fabrication of a surgical guide. For practitioners unfamiliar with digital workflows, these steps may initially appear complex or time-consuming. However, studies have shown that guided procedures can improve clinical efficiency by reducing procedural difficulties and facilitating faster canal localization, thereby streamlining treatment compared with traditional approaches [25, 26].

The majority of participants expressed willingness to use GEA in their future clinical practice, representing a positive finding of this study. This suggests that many dentists recognise the potential benefits of guided endodontic technology and are open to incorporating it into clinical care. Ongoing advancements in digital dentistry—particularly in additive manufacturing, digital imaging, and artificial intelligence—are expected to further improve the accessibility and efficiency of guided endodontic systems. These developments may help reduce costs, streamline clinical workflows, and ultimately support broader integration of guided techniques into routine dental practice [22, 29].

The study also identified significant associations between practitioners’ awareness and knowledge of GEA and their demographic characteristics. Clinicians with greater professional experience and higher academic qualifications reported higher levels of awareness, knowledge, and training in guided endodontic techniques. This may be explained by cumulative clinical experience, as more experienced practitioners are often better able to recognize the limitations of conventional techniques and the advantages of emerging technologies [30, 31].

Furthermore, dentists with postgraduate qualifications or specialized training are more likely to be exposed to innovative endodontic techniques through research involvement, professional networks, and continuing education programs. These findings are consistent with previous studies showing that clinicians with higher levels of education or specialized training are generally more receptive to adopting new technologies [21, 30–32]. This greater willingness may be attributed to increased familiarity with evidence-based practice, improved access to advanced clinical infrastructure, and enhanced ability to critically evaluate and implement technological innovations [21, 32].

Participants also expressed strong support for integrating guided endodontics into dental education and training programs. Most respondents indicated that GEA should be included at both undergraduate and postgraduate levels. This perspective aligns with the ongoing shift in dental education toward digitally enabled learning environments and technology-driven clinical training, which can better prepare dental practitioners to effectively adopt and apply these techniques in clinical practice [29].

Several limitations should be considered when interpreting the findings of this study. First, the cross-sectional design limits the ability to assess changes in knowledge, attitudes, and adoption over time. Second, the use of an online survey may have introduced selection bias, as it may have attracted respondents more interested in digital dentistry. Third, self-reported data may be subject to response bias. It should also be noted that this study did not involve clinical intervention; therefore, no direct ethical concerns related to patient care were present. Although associations between clinical experience and training were explored, the study did not allow detailed stratification based on both experience and specialty simultaneously. Future studies should consider more detailed subgroup analyses to better understand the interaction between experience, training, and adoption of guided endodontic access. Future research should also include objective clinical assessments and longitudinal study designs to provide a more comprehensive evaluation of the implementation and effectiveness of guided endodontic techniques in clinical practice. As digital dentistry continues to evolve, guided endodontic techniques are expected to play an increasingly important role in improving treatment accuracy, reducing procedural errors, and enhancing clinical outcomes in endodontic practice [32]. procedural mistakes, and improving clinical outcomes in endodontic practice [32].

## Conclusions

This study demonstrates that dental practitioners in Saudi Arabia generally have a high level of awareness of GEA and recognize its potential benefits, particularly in improving procedural accuracy, enhancing treatment safety, and facilitating the management of complex endodontic cases. However, several practical barriers—including cost, workflow complexity, and the need for specialized training—continue to limit its widespread adoption. Addressing these challenges through targeted educational initiatives, ongoing technological advancements, and integration of digital workflows into dental curricula may promote broader implementation of GEA. Such efforts may ultimately contribute to improved treatment outcomes and the advancement of minimally invasive endodontic practice.

## Supplementary Information

Supplementary Material 1.

Supplementary Material 2.

Supplementary Material 3.

Supplementary Material 4.

## Data Availability

The datasets created and/or analyzed for the current study are not publicly accessible because ethics approval was given on the grounds that only the researchers involved in the study would have access to the identified data, but they are available from the corresponding author upon justifiable request.
