# Supplementary material for: Knowledge, attitudes, and perceived barriers toward guided endodontic access among dental practitioners in Saudi Arabia: a cross-sectional study
Source: BMC Oral Health. 2026 Jun 4;26:1571. doi: 10.1186/s12903-026-08743-3 (PMC13505114; doi:10.1186/s12903-026-08743-3)
Supplement: Supplementary file 1 [file 12903_2026_8743_MOESM1_ESM.docx]

**1. Do you agree to participate voluntarily in this research study?**
○ Agree
○ Do not agree

## **Section 2: Demographic and Professional Characteristics**

**Gender**
○ Male
○ Female

**Years of Clinical Experience**
○ 1–5 years
○ 6–10 years
○ 11–15 years
○ >15 years

**Qualification**
○ Undergraduate
○ Postgraduate / Board-certified
○ Workshop / Continuing education

## **Section 3: Clinical Practice**

**Q4. Do you routinely perform endodontic treatments?**
○ Yes
○ No

## **Section 4: Awareness of Guided Endodontic Access (GEA)**

**Q5. Have you heard of “guided endodontic access (GEA)”?**
○ Yes
○ No
○ Unsure

**Q6. How would you describe guided endodontic access?**
○ CBCT + 3D-printed guide
○ Computer-controlled access
○ Manual technique
○ Unsure

## **Section 5: Training and Experience**

**Q7. Have you received any formal training (lecture, workshop, or course) about guided endodontic access?**
○ Yes
○ No

## **Section 6: Knowledge of GEA**

**Q8. Rate your self-perceived level of knowledge about guided endodontic access:**
○ Very poor
○ Poor
○ Moderate
○ Good
○ Excellent

## **Section 7: Attitudes and Perceptions (Likert Scale)**

**For the following statements, please indicate your level of agreement:**
○ Strongly disagree
○ Disagree
○ Neutral
○ Agree
○ Strongly agree

**Q9. GEA improves the accuracy and safety of canal location.**

**Q10. GEA should be used routinely in complex or calcified cases.**

**Q11. The cost and time required for GEA limit its practical application.**

**Q12. I would be willing to adopt GEA if proper training and equipment were available.**

**Q13. Guided access represents a major advancement in endodontic practice.**

**Q14. I believe undergraduate or continuing education programs should include training on GEA**

## **Section 8: Adoption Intention**

**Q15. Do you plan to adopt guided endodontic access in your clinical practice within the next 2 years?**
○ Yes
○ Unsure
○ No
○ Already implemented
