# Supplementary material for: Knowledge, attitudes, and perceived barriers toward guided endodontic access among dental practitioners in Saudi Arabia: a cross-sectional study
Source: BMC Oral Health. 2026 Jun 4;26:1571. doi: 10.1186/s12903-026-08743-3 (PMC13505114; doi:10.1186/s12903-026-08743-3)
Supplement: Supplementary file 2 [file 12903_2026_8743_MOESM2_ESM.docx]

S 2. Association Between Gender and Awareness, Perceptions, and Adoption of Guided Endodontic Access (N = 401)

| **Variable** | **Category** | **Male n (%) (n=198)** | **Female n (%) (n=203)** | **χ² (df)** | **p-value** |
| --- | --- | --- | --- | --- | --- |
| Q.4 Do you routinely perform endodontic treatments? | Yes | 180 (44.9%) | 170 (42.4%) | 14.17 | **.007** |
|  | No | 18 (4.9%) | 33 (8.2%) |  |  |
| Q5. Have you heard of “guided endodontic access (GEA)”? | Yes | 181 (45.1%) | 162 (40.4%) | 11.61 | **.021** |
|  | No | 10 (2.5%) | 19 (4.7%) |  |  |
|  | Unsure | 7 (1.7%) | 22 (5.5%) |  |  |
| Q6. How would you describe guided endodontic access? | CBCT + 3D printed guide | 172 (42.7%) | 164 (40.8%) | 8.96 | .346 |
|  | Computer controlled access | 17 (4.2%) | 15 (3.7%) |  |  |
|  | Manual technique | 5 (1.2%) | 9 (2.2%) |  |  |
|  | Unsure | 4 (1.0%) | 15 (3.7%) |  |  |
| Q7. Have you received any formal training (lecture, workshop, or course) about guided endodontic access? | Yes | 167 (41.6%) | 152 (37.9%) | 11.41 | **.022** |
|  | No | 31 (7.7%) | 51 (12.7%) |  |  |
| Q8. Rate your self-perceived level of knowledge about guided endodontic access: | Very poor | 7 (1.7%) | 5 (1.2%) | 26.42 | **.003** |
|  | Poor | 9 (2.2%) | 27 (6.7%) |  |  |
|  | Moderate | 35 (8.7%) | 50 (12.4%) |  |  |
|  | Good | 85 (21.4%) | 74 (18.4%) |  |  |
|  | Excellent | 62 (15.5%) | 47 (11.7%) |  |  |
| Q9. GEA improves the accuracy and safety of canal location. | Strongly disagree | 3 (0.7%) | 3 (0.7%) | 3.09 | .928 |
|  | Disagree | 2 (0.5%) | 1 (0.2%) |  |  |
|  | Neutral | 22 (5.5%) | 29 (7.2%) |  |  |
|  | Agree | 94 (23.4%) | 90 (22.4%) |  |  |
|  | Strongly agree | 77 (19.2%) | 80 (20.0%) |  |  |
| Q10. GEA should be used routinely in complex or calcified cases. | Strongly disagree | 3 (0.7%) | 5 (1.2%) | 12.41 | .259 |
|  | Disagree | 5 (1.2%) | 7 (1.7%) |  |  |
|  | Neutral | 28 (7.0%) | 40 (10.0%) |  |  |
|  | Agree | 112 (27.9%) | 86 (21.4%) |  |  |
|  | Strongly agree | 50 (12.5%) | 65 (16.2%) |  |  |
| Q11. The cost and time required for GEA limit its practical application. | Strongly disagree | 1 (0.2%) | 3 (0.7%) | 7.03 | .723 |
|  | Disagree | 4 (1.0%) | 5 (1.2%) |  |  |
|  | Neutral | 39 (9.7%) | 47 (11.7%) |  |  |
|  | Agree | 96 (23.9%) | 103 (25.7%) |  |  |
|  | Strongly agree | 58 (14.4%) | 45 (11.2%) |  |  |
| Q12. I would be willing to adopt GEA if proper training and equipment were available. | Strongly disagree | 4 (1%) | 2 (0.5%) | 7.73 | .656 |
|  | Disagree | 5 (1.2%) | 8 (2.0%) |  |  |
|  | Neutral | 30 (7.5%) | 42 (10.5%) |  |  |
|  | Agree | 103 (25.7%) | 101 (25.2%) |  |  |
|  | Strongly agree | 56 (14.0%) | 50 (12.5%) |  |  |
| Q13. Guided access represents a major advancement in endodontic practice. | Strongly disagree | 3 (0.7%) | 2 (0.5%) | 4.62 | .915 |
|  | Disagree | 5 (1.2%) | 5 (1.2%) |  |  |
|  | Neutral | 42 (10.5%) | 45 (11.2%) |  |  |
|  | Agree | 100 (24.9%) | 104 (25.9%) |  |  |
|  | Strongly agree | 48 (12.0%) | 47 (11.7%) |  |  |
| Q14. I believe undergraduate or continuing education programs should include training on GEA | Strongly disagree | 0 (0.0%) | 0 (0.0%) | 15.90 | **.044** |
|  | Disagree | 4 (1.0%) | 4 (1.0%) |  |  |
|  | Neutral | 18 (4.5%) | 42 (10.5%) |  |  |
|  | Agree | 104 (25.9%) | 94 (23.4%) |  |  |
|  | Strongly agree | 72 (17.9%) | 63 (15.7%) |  |  |
| Q15. Do you plan to adopt guided endodontic access in your clinical practice within the next 2 years? | Yes | 151 (37.7%) | 156 (38.9%) | 1.59 | .991 |
|  | Unsure | 25 (6.2%) | 24 (6.0%) |  |  |
|  | No | 11 (2.7%) | 14 (3.5%) |  |  |
|  | Already implemented | 11 (2.7%) | 9 (2.2%) |  |  |
| Data are presented as frequencies and percentages. Associations were evaluated using the chi-square (χ²) test. Statistically significant p-values (p < 0.05) are indicated in bold. | | | | | |
