# Supplementary material for: Knowledge, attitudes, and perceived barriers toward guided endodontic access among dental practitioners in Saudi Arabia: a cross-sectional study
Source: BMC Oral Health. 2026 Jun 4;26:1571. doi: 10.1186/s12903-026-08743-3 (PMC13505114; doi:10.1186/s12903-026-08743-3)
Supplement: Supplementary file 3 [file 12903_2026_8743_MOESM3_ESM.docx]

S 3. Association Between Years of Experience and Awareness, Perceptions, and Adoption of Guided Endodontic Access (N = 401)

| **Variable** | **Category** | **1–5 years n (%) (n=217)** | **6–10 years n (%) (n=155)** | **11–15 years n (%) (n=26)** | **>15 years n (%) (n=3)** | **χ² (df)** | **p-value** |
| --- | --- | --- | --- | --- | --- | --- | --- |
| Q.4 Do you routinely perform endodontic treatments? | Yes | 177 (44.1%) | 147 (36.7%) | 23 (5.7%) | 3 (0.7%) | 15.88 | **.044** |
|  | No | 40 (10.0%) | 8 (2.0%) | 3 (0.7%) | 0 (0.0%) |  |  |
| Q5. Have you heard of “guided endodontic access (GEA)”? | Yes | 171 (42.6%) | 146 (36.4%) | 24 (6.0%) | 2 (0.5%) | 25.95 | **.001** |
|  | No | 21 (5.2%) | 6 (1.5%) | 2 (0.5%) | 0 (0.0%) |  |  |
|  | Unsure | 25 (6.2%) | 3 (0.7%) | 0 (0.0%) | 1 (0.2%) |  |  |
| Q6. How would you describe guided endodontic access? | CBCT + 3D printed guide | 173 (43.1%) | 141 (35.2%) | 21 (5.2%) | 1 (0.2%) | 24.14 | .086 |
|  | Computer controlled access | 17 (4.2%) | 10 (2.5%) | 4 (1.0%) | 1 (0.2%) |  |  |
|  | Manual Technique | 13 (3.2%) | 1 (0.2%) | 0 (0.0%) | 0 (0.0%) |  |  |
|  | Unsure | 14 (3.5%) | 3 (0.7%) | 1 (0.2%) | 1 (0.2%) |  |  |
| Q7. Have you received any formal training (lecture, workshop, or course) about guided endodontic access? | Yes | 149 (36.7%) | 143 (35.6%) | 24 (6.0%) | 3 (0.7%) | 38.06 | **.000** |
|  | No | 68 (17.0%) | 12 (3.0%) | 2 (0.5%) | 0 (0.0%) |  |  |
| Q8. Rate your self-perceived level of knowledge about guided endodontic access: | Very poor | 11 (2.7%) | 1 (0.2%) | 0 (0.0%) | 0 (0.0%) | 37.57 | **.010** |
|  | Poor | 28 (7.0%) | 8 (2.0%) | 0 (0.0%) | 0 (0.0%) |  |  |
|  | Moderate | 48 (12.0%) | 29 (7.2%) | 5 (1.2%) | 3 (0.7%) |  |  |
|  | Good | 81 (20.2%) | 67 (16.7%) | 11 (2.7%) | 0 (0.0%) |  |  |
|  | Excellent | 49 (12.2%) | 50 (12.5%) | 10 (2.5%) | 0 (0.0%) |  |  |
| Q9. GEA improves the accuracy and safety of canal location. | Strongly disagree | 4 (1.0%) | 1 (0.2%) | 0 (0.0%) | 1 (0.2%) | 15.38 | .497 |
|  | Disagree | 1 (0.2%) | 0 (0.0%) | 0 (0.0%) | 2 (0.5%) |  |  |
|  | Neutral | 34 (8.5%) | 15 (3.7%) | 2 (0.5%) | 0 (0.0%) |  |  |
|  | Agree | 89 (22.2%) | 77 (19.2%) | 18 (4.5%) | 0 (0.0%) |  |  |
|  | Strongly agree | 89 (22.2%) | 62 (15.5%) | 6 (1.5%) | 0 (0.0%) |  |  |
| Q10. GEA should be used routinely in complex or calcified cases. | Strongly disagree | 5 (1.2%) | 2 (0.5%) | 1 (0.2%) | 0 (0.0%) | 35.41 | **.018** |
|  | Disagree | 7 (1.7%) | 2 (0.5%) | 2 (0.5%) | 1 (0.2%) |  |  |
|  | Neutral | 44 (11.0%) | 24 (6.0%) | 0 (0.0%) | 0 (0.0%) |  |  |
|  | Agree | 95 (23.7%) | 86 (21.4%) | 15 (3.7%) | 2 (0.5%) |  |  |
|  | Strongly agree | 66 (16.5%) | 41 (10.2%) | 8 (2.0%) | 0 (0.0%) |  |  |
| Q11. The cost and time required for GEA limit its practical application. | Strongly disagree | 2 (0.5%) | 1 (0.2%) | 1 (0.2%) | 0 (0.0%) | 18.95 | .525 |
|  | Disagree | 5 (1.2%) | 3 (0.7%) | 1 (0.2%) | 0 (0.0%) |  |  |
|  | Neutral | 50 (12.5%) | 31 (7.7%) | 5 (1.2%) | 0 (0.0%) |  |  |
|  | Agree | 98 (24.4%) | 88 (21.9%) | 12 (3.0%) | 1 (0.2%) |  |  |
|  | Strongly agree | 62 (15.5%) | 32 (8.0%) | 7 (1.7%) | 2 (0.5%) |  |  |
| Q12. I would be willing to adopt GEA if proper training and equipment were available. | Strongly disagree | 4 (1.0%) | 1 (0.2%) | 0 (0.0%) | 1 (0.2%) | 72.18 | **.000** |
|  | Disagree | 4 (1.0%) | 6 (1.5%) | 1 (0.2%) | 2 (0.5%) |  |  |
|  | Neutral | 41 (10.2%) | 28 (7.0%) | 3 (0.7%) | 0 (0.0%) |  |  |
|  | Agree | 108 (26.9%) | 82 (20.4%) | 14 (3.5%) | 0 (0.0%) |  |  |
|  | Strongly agree | 60 (15.0%) | 38 (9.5%) | 8 (2.0%) | 0 (0.0%) |  |  |
| Q13. Guided access represents a major advancement in endodontic practice. | Strongly disagree | 3 (0.7%) | 1 (0.2%) | 1 (0.2%) | 0 (0%) | 27.96 | .110 |
|  | Disagree | 3 (0.7%) | 5 (1.2%) | 1 (0.2%) | 1 (0.2%) |  |  |
|  | Neutral | 47 (11.7%) | 33 (8.2%) | 6 (1.5%) | 1 (0.2%) |  |  |
|  | Agree | 109 (27.2%) | 82 (20.4%) | 12 (3.0%) | 1 (0.2%) |  |  |
|  | Strongly agree | 55 (13.7%) | 34 (8.5%) | 6 (1.5%) | 0 (0.0%) |  |  |
| Q14. I believe undergraduate or continuing education programs should include training on GEA | Strongly disagree | 0 (0.0%) | 0 (0.0%) | 0 (0.0%) | 0 (0.0%) | 24.12 | .087 |
|  | Disagree | 6 (1.5%) | 1 (0.2%) | 1 (0.2%) | 0 (0.0%) |  |  |
|  | Neutral | 38 (9.5%) | 16 (4.0 %) | 5 (1.2%) | 1 (0.2%) |  |  |
|  | Agree | 95 (23.7%) | 92 (22.9%) | 11 (2.7%) | 0 (0.0%) |  |  |
|  | Strongly agree | 78 (19.5%) | 46 (11.5%) | 9 (2.2%) | 2 (0.5%) |  |  |
| Q15. Do you plan to adopt guided endodontic access in your clinical practice within the next 2 years? | Yes | 149 (37.2%) | 136 (33.9%) | 22 (5.5%) | 0 (0.0%) | 36.42 | **.003** |
|  | Unsure | 37 (9.2%) | 9 (2.2%) | 1 (0.2%) | 2 (0.5%) |  |  |
|  | No | 19 (4.7%) | 3 (0.7%) | 2 (0.5%) | 1 (0.2%) |  |  |
|  | Already implemented | 12 (3.0%) | 7 (1.7%) | 1 (0.2%) | 0 (0.0%) |  |  |
| Data are presented as frequencies and percentages. Associations were evaluated using the chi-square (χ²) test. Statistically significant p-values (p < 0.05) are indicated in bold. | | | | | | | |
