# Supplementary material for: Knowledge, attitudes, and perceived barriers toward guided endodontic access among dental practitioners in Saudi Arabia: a cross-sectional study
Source: BMC Oral Health. 2026 Jun 4;26:1571. doi: 10.1186/s12903-026-08743-3 (PMC13505114; doi:10.1186/s12903-026-08743-3)
Supplement: Supplementary file 4 [file 12903_2026_8743_MOESM4_ESM.docx]

S 4. Association Between Qualification and Awareness, Perceptions, and Adoption of Guided Endodontic Access (N = 401)

| **Variable** | **Category** | **Undergraduate n (%) (n=215)** | **Postgraduate/ Board Member n (%) (n=166)** | **Workshop/ Cont. Education n (%) (n=20)** | **χ² (df)** | **p-value** |
| --- | --- | --- | --- | --- | --- | --- |
| Q.4 Do you routinely perform endodontic treatments? | Yes | 183 (45.6%) | 154 (38.4%) | 13 (3.2%) | 15.56 | **.004** |
|  | No | 32 (8.0%) | 12 (2.7%) | 7 (1.7%) |  |  |
| Q5. Have you heard of “guided endodontic access (GEA)”? | Yes | 182 (45.4%) | 153 (38.2%) | 8 (2.0%) | 63.76 | **.000** |
|  | No | 17 (4.2%) | 10 (2.5%) | 2 (0.5%) |  |  |
|  | Unsure | 16 (4.0%) | 3 (0.7%) | 10 (2.5%) |  |  |
| Q6. How would you describe guided endodontic access? | CBCT + 3D printed guide | 179 (44.6%) | 146 (36.4%) | 11 (2.7%) | 31.84 | **.000** |
|  | Computer controlled access | 16 (4.0%) | 13 (3.2%) | 3 (0.7%) |  |  |
|  | Manual technique | 6 (1.5%) | 7 (1.7%) | 1 (0.2%) |  |  |
|  | Unsure | 14 (3.5%) | 0 (0.0%) | 5 (1.2%) |  |  |
| Q7. Have you received any formal training (lecture, workshop, or course) about guided endodontic access? | Yes | 162 (40.3%) | 147 (36.7%) | 10 (2.5%) | 25.08 | **.000** |
|  | No | 53 (13.2%) | 19 (4.7%) | 10 (2.5%) |  |  |
| Q8. Rate your self-perceived level of knowledge about guided endodontic access: | Very poor | 8 (2.0%) | 1 (0.2%) | 3 (0.7%) | 35.12 | **.000** |
|  | Poor | 25 (6.2%) | 7 (1.7%) | 4 (1.0%) |  |  |
|  | Moderate | 40 (10.0%) | 39 (9.7%) | 6 (1.5%) |  |  |
|  | Good | 77 (19.2%) | 78 (19.5%) | 4 (1.0%) |  |  |
|  | Excellent | 65 (16.2%) | 41 (10.2%) | 3 (0.7%) |  |  |
| Q9. GEA improves the accuracy and safety of canal location. | Strongly disagree | 4 (1.0%) | 2 (0.5%) | 0 (0.0%) | 10.90 | .208 |
|  | Disagree | 3 (0.7%) | 0 (0.0%) | 0 (0.0%) |  |  |
|  | Neutral | 32 (8.0%) | 15 (3.7%) | 4 (1.0%) |  |  |
|  | Agree | 85 (21.2%) | 90 (22.4%) | 9 (2.2%) |  |  |
|  | Strongly agree | 91 (22.7%) | 59 (14.7%) | 7 (1.7%) |  |  |
| Q10. GEA should be used routinely in complex or calcified cases. | Strongly disagree | 4 (1.0%) | 3 (0.7%) | 1 (0.2%) | 36.39 | **.000** |
|  | Disagree | 4 (1.0%) | 4 (1.0%) | 4 (1.0%) |  |  |
|  | Neutral | 35 (8.7%) | 28 (7.0%) | 5 (1.2%) |  |  |
|  | Agree | 101 (25.2%) | 90 (22.4%) | 7 (1.7%) |  |  |
|  | Strongly agree | 71 (17.7%) | 41 (10.2%) | 3 (0.7%) |  |  |
| Q11. The cost and time required for GEA limit its practical application. | Strongly disagree | 2 (0.5%) | 1 (0.2%) | 1 (0.2%) | 10.92 | .364 |
|  | Disagree | 5 (1.2%) | 3 (0.7%) | 1 (0.2%) |  |  |
|  | Neutral | 48 (12.0%) | 31 (7.5%) | 7 (1.7%) |  |  |
|  | Agree | 106 (26.4%) | 88 (21.2%) | 5 (1.2%) |  |  |
|  | Strongly agree | 54 (13.5%) | 43 (10.7%) | 6 (1.5%) |  |  |
| Q12. I would be willing to adopt GEA if proper training and equipment were available. | Strongly disagree | 4 (1.0%) | 1 (0.2%) | 1 (0.2%) | 12.61 | .246 |
|  | Disagree | 5 (1.2%) | 7 (1.7%) | 1 (0.2%) |  |  |
|  | Neutral | 36 (9.0%) | 29 (7.2%) | 7 (1.7%) |  |  |
|  | Agree | 113 (28.2%) | 85 (21.2%) | 6 (1.5%) |  |  |
|  | Strongly agree | 57 (14.2%) | 44 (11.2%) | 5 (1.2%) |  |  |
| Q13. Guided access represents a major advancement in endodontic practice. | Strongly disagree | 3 (0.7%) | 1 (0.2%) | 1 (0.2%) | 42.40 | **.000** |
|  | Disagree | 2 (0.5%) | 5 (1.2%) | 3 (0.7%) |  |  |
|  | Neutral | 42 (10.5%) | 39 (9.7%) | 6 (1.5%) |  |  |
|  | Agree | 115 (28.7%) | 85 (21.2%) | 4 (1.0%) |  |  |
|  | Strongly agree | 53 (13.2%) | 36 (9.0%) | 6 (1.5%) |  |  |
| Q14. I believe undergraduate or continuing education programs should include training on GEA | Strongly disagree | 0 (0%) | 0 (0%) | 0 (0%) | 6.20 | .625 |
|  | Disagree | 4 (1.0%) | 3 (0.7%) | 1 (0.2%) |  |  |
|  | Neutral | 28 (7.0%) | 29 (7.2%) | 3 (0.7%) |  |  |
|  | Agree | 108 (26.9%) | 83 (20.7%) | 7 (1.7%) |  |  |
|  | Strongly agree | 75 (18.7%) | 51 (12.7%) | 9 (2.2%) |  |  |
| Q15. Do you plan to adopt guided endodontic access in your clinical practice within the next 2 years? | Yes | 157 (39.2%) | 142 (35.4%) | 8 (2.0%) | 36.90 | **.000** |
|  | Unsure | 35 (8.7%) | 11 (2.7%) | 3 (0.7%) |  |  |
|  | No | 14 (3.5%) | 5 (1.2%) | 6 (1.5%) |  |  |
|  | Already implemented | 9 (2.2%) | 8 (2.0%) | 3 (0.7%) |  |  |
| Data are presented as frequencies and percentages. Associations were evaluated using the chi-square (χ²) test. Statistically significant p-values (p < 0.05) are indicated in bold. | | | | | | |
